# Home Management of Heart Failure and Arrhythmias in Patients with Cardiac Devices during Pandemic

**DOI:** 10.3390/jcm10081618

**Published:** 2021-04-11

**Authors:** Andrea Matteucci, Michela Bonanni, Marco Centioni, Federico Zanin, Francesco Geuna, Gianluca Massaro, Giuseppe Sangiorgi

**Affiliations:** 1 Department of Experimental Medicine, University of Rome “Tor Vergata”, 00133 Rome, Italy; michelabonanni91@gmail.com (M.B.); marco.centioni@gmail.com (M.C.); federicozanin89@gmail.com (F.Z.); francesco.geuna@ptvonline.it (F.G.); 2 Division of Cardiology, University Hospital “Tor Vergata”, 00133 Rome, Italy; gianluca88massaro@gmail.com; 3 Department of Biomedicine and Prevention, Tor Vergata University, 00133 Rome, Italy; gsangiorgi@gmail.com

**Keywords:** remote monitoring, heart failure, arrhythmias, COVID-19

## Abstract

Background: The in-hospital management of patients with cardiac implantable electronic devices (CIEDs) changed early in the COVID-19 pandemic. Routine in-hospital controls of CIEDs were converted into remote home monitoring (HM). The aim of our study was to investigate the impact of the lockdown period on CIEDs patients and its influence on in-hospital admissions through the analysis of HM data. Methods: We analysed data recorded from 312 patients with HM during the national quarantine related to COVID-19 and then compared data from the same period of 2019. Results: We observed a reduction in the number of HM events in 2020 when compared to 2019. Non-sustained ventricular tachycardia episodes decreased (18.3% vs. 9.9% *p* = 0.002) as well as atrial fibrillation episodes (29.2% vs. 22.4% *p* = 0.019). In contrast, heart failure (HF) alarm activation was lower in 2019 than in 2020 (17% vs. 25.3% *p* = 0.012). Hospital admissions for critical events recorded with CIEDs dropped in 2020, including those for HF. Conclusions: HM, combined with telemedicine use, has ensured the surveillance of CIED patients. In 2020, arrhythmic events and hospital admissions decreased significantly compared to 2019. Moreover, in 2020, patients with HF arrived in hospital in a worse clinical condition compared to previous months.

## 1. Introduction

The COVID-19 disease has rapidly become a great challenge in every medical field worldwide, including more difficult access to care for patients with chronic illnesses. Many studies showed a strong relationship between cardiovascular disease (CVD) and COVID-19 as many COVID-19 patients were already affected by or developed a CVD during the infection [1,2].

Patients with cardiac implantable electronic devices (CIEDs) require periodic outpatient visits to monitor the proper function of the devices, patient condition and to record any critical events. In order to safeguard patients’ health, during the early stages of the pandemic, the Heart Rhythm Society (HRS) issued guidelines encouraging the conversion of all routine in-hospital CIEDs controls into remote home monitoring (HM) [3]. HM is as effective as, or even more efficient, than routine in-office visits to detect the early onset of acute events due to their quick transmission to physicians [4].

On 9 March 2020, the Italian Government declared a state of emergency, issuing strict social containment measures. Hospitals established infection emergency protocols and many outpatient appointments, including those related to CIED assessment, were postponed during the entire lockdown period. This extraordinary situation confirmed the importance of HM, which allowed for good patient follow-up and avoiding in-office evaluations.

Several cardiovascular centres showed that early in the COVID-19 pandemic hospitalizations for acute coronary syndromes (ACS) were reduced [5,6,7,8]. The impact of the restrictive measures on CIEDs patients and their hospitalizations was not explored. The aim of our study is to show the benefits and disadvantages of the social restrictions imposed on patients receiving CIEDs and which factors have most influenced hospital admissions through the analysis of remote monitoring during the lockdown period for COVID-19. 

## 2. Materials and Methods

We followed CIEDs patients in the Cardiology Department of “Policlinico Tor Vergata” of Rome in this observational study. All the patients enrolled had remote home monitoring. We analysed the data recorded by HM during the national state of emergency related to COVID-19. Specifically, we analysed data recorded from 9 March to 4 May 2020 and compared them to the control period between 9 March to 4 May 2019. Inclusion criteria were: (1) the implantation of the device must have been before 2019, (2) the patients had active (daily or weekly) transmissions in both 2019 and 2020, (3) the use of the remote monitoring web site reviewed by our centre. Consequently, exclusion criteria were: (1) patients without periodical transmission or transferred to other centres, (2) CIEDs where it was not possible to discriminate ventricular events with accuracy (e.g., single-chamber pacemakers), (3) devices with only a monitoring function where no programming was available in the case of events (e.g., implantable loop recorders). In addition, we recorded hospitalizations in the same patients that occurred during the lockdown period of 2020, and the control period of 2019. Demographic, clinical and instrumental data were obtained from the in-hospital records. We utilized the chronic kidney disease epidemiology collaboration (CKD–EPI) equation to calculate patient’s glomerular filtration rate (GFR) and we considered the presence of chronic kidney disease for the value of GFR inferior to 60 mL/min/m^2^. The outcome parameters were extracted from remote monitoring data collected in both 2020 and 2019. We stored data of every automatic weekly transmission of patients with cardiac resynchronization therapy (CRT), implantable cardioverter defibrillator (ICD) and pacemaker (PM), which were received and presented as a review in specific web sites. Critical events were transmitted separately and flagged for attention. Recorded events include out-of-range impedance, atrial fibrillation (AF), ventricular fibrillation (VF), non-sustained and sustained ventricular tachycardia (NSVT, SVT), anti-tachycardia pacing therapy (ATP), appropriate and inappropriate shocks, pacemaker-mediated tachycardia (PMT), heart failure alarm activation, elective replacement indicator (ERI) battery alarm, atrial and ventricular lead noise alert. Emphasis has been given to heart failure (HF) alarm activation. We recorded the data using the CIEDs algorithms (Medtronic OptiVol™ 2.0 Fluid Status Monitoring, Abbott CorVue™ congestion monitoring, Boston Scientific HeartLogic™ Heart Failure Diagnostic, Biotronik Heart Failure Monitor™). Due to the different HF detection systems in multiple CIED manufacturers, we also conducted telephone interviews with patients with a HF alarm to correlate the engineering data with clinical symptoms. All patients provided consent in their medical records to disclose data in an anonymous form for research purposes.

### Statistical Analysis

We performed a statistical analysis using IBM SPSS Statistics 26. Categorical variables are expressed as counts and percentages; continuous variables are expressed as mean ± standard deviation. We used the Shapiro–Wilk test to check the normality of data samples. Continuous variables were compared using a *t*-test for paired samples. Categorical variables were compared using the McNemar test, with Holm–Bonferroni adjustment for the p-value. Logistic regression identified predictors of hospitalization in 2019 and 2020. A valid model was not developed for 2020 due to the low number of hospital admissions. The univariate predictors with *p* < 0.05 and greatest clinical utility were selected for subsequent multivariate analysis, as allowed by our sample size.

## 3. Results

A total of 312 patients were enrolled. All of the patients had CIEDs. Of the 312 patients, 185 (59.3%) had PM, while 127 (40.7%) had ICD or CRT. Demographic characteristics and clinical features are summarized in Table 1. 

The mean age of the population studied was 71 ± 7.2 years and 48% of the patients were men. A considerable number of patients had the common cardiovascular risk factors. Specifically, 194 (62.1%) patients had hypertension, 87 (27.9%) had diabetes and 63 (20%) had chronic kidney disease. In addition, 56 patients (18%) had chronic obstructive pulmonary disease. Furthermore, 104 (33.3%) patients had chronic AF and received treatment with anticoagulants. At the time of the analysis all patients were treated with optimal medical therapy. In particular, 262 (84%) were on angiotensin receptor blockers (ARB), or angiotensin converting enzyme inhibitors (ACEI), or angiotensin receptor neprilysin inhibitors (ARNI), 158 (50.6%) were on beta blockers, 106 (33.6%) were on aldosterone antagonists, 141 (45.2%) were on diuretics, 59 (18.9%) were on antiarrhythmic drugs, 12 (3.8%) were on digoxin and 123 (39.4%) were on statins. Table 1 shows the echocardiographic characteristics of the patients. The mean ejection fraction was 43.5% ± 9.8, valve disease, including valve stenosis or valve regurgitation of moderate (++) or higher grade, diastolic function was assessed with a combination of parameters, according to the recommendations of the American Society of Echocardiography.

**Figure 1:**
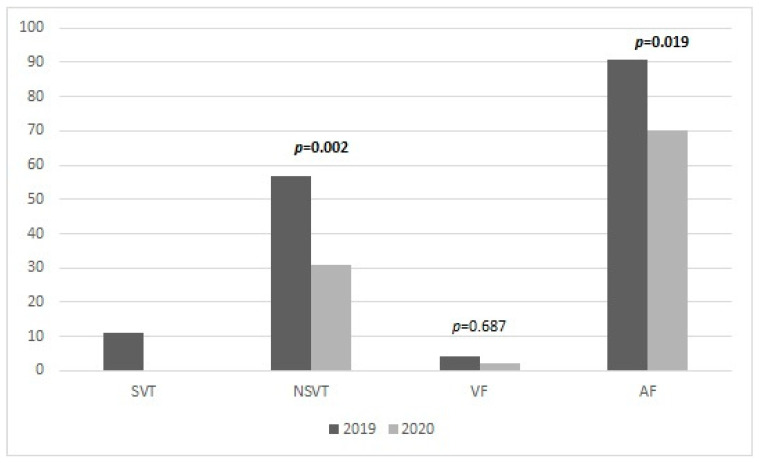
Histogram of ventricular arrhythmia and atrial fibrillation episode occurrence. Comparison between 2019 and 2020. SVT = sustained ventricular tachycardia, NSVT = non-sustained ventricular tachycardia, VF = ventricular fibrillation, AF = atrial fibrillation.

From 9 March to 4 May 2020, we observed a significant reduction in the number of remote monitoring events, when compared to the same period of 2019. In particular, 57 (18.3%) episodes of NSVT arrhythmia were recorded, compared with 31 (9.9%) episodes recorded in 2020 (*p* = 0.002). In addition, in 2019, patients developed more AF events, compared with 2020 (29.2% vs. 22.4% *p* = 0.019) (Figure 1). 

In contrast, HF alarm activation was lower in 2019 than in 2020 (17% vs. 25.3% *p* = 0.012). It is pivotal to note that the hospitalization related to critical events recorded from HM were significantly reduced in the lockdown period of 2020 compared to the same period of 2019 (6.4% vs. 0.6% *p* < 0.001) (Table 2). In fact, during the study period we recorded only two hospital admissions, compared to 20 in the same period in 2019 (*p* < 0.001). The first hospitalization in 2020 was for an episode of VF, while the second one was for severe HF in a CRT-D patient. 

Results from the logistic regression are summarized in Table 3. The major predictor of hospitalization in 2019 was the ERI battery alarm (OR = 827.5 CI = 46.7–144654.9 *p* < 0.001). Other significant predictors of hospitalization were VF (OR = 262.4 CI 11.3–6114.3 *p* = 0.001), ventricular lead noise alert (OR = 66.909 CI = 6.880–650.665 *p* = 0.001), followed by SVT (OR = 39.3 CI 4.5–339.9 *p* = 0.001) and atrial lead noise alert (OR = 13.138 CI = 1.318–130.942 *p* = 0.028). 

## 4. Discussion

To fight the COVID-19 pandemic, health care systems have been reorganized and restrictive measures have been adopted in order to contain the infection. In particular, outpatient visits have been significantly reduced and postponed to decrease contagion. As a result, there has been a considerable delay in long-planned in-office visits. In this analysis, we focused on outpatient management of CIEDs patients with HM.

Due to the pandemic outbreak, our hospital became a hub for patients with COVID-19 and in-office visit management of patients with implantable cardiac devices was developed into telemedicine with HM [3]. Through remote HM, it was possible to maintain nearly continuous arrhythmias and device surveillance to quickly identify patients requiring attention (62% of pts of the total cohort). This tool gave us the opportunity to avoid in-hospital evaluations of a large number of patients with stable parameters at the tele monitoring data analysis. In particular, it was possible to remotely control cardiology patients at high risk of developing a severe form of COVID-19. In order to follow as many patients as possible, we provided remote monitoring to all critical patients who did not have it. In this way, during the lockdown period we remotely control more than 500 patients with cardiac devices. Using data recorded from the HM, we analysed critical events (e.g., HF alarms, compromised device integrity, arrhythmia onset) in order to investigate their impact on hospital admissions in patients with CIEDs. We observed a significant decrease in the number of emergency department/urgent visits for critical events detected by HM during the study period. In fact, we recorded only two hospital admissions, the first for an episode of VF and the second for severe HF in a CRT-D patient. In 2019, VF was one of the major predictors of hospitalization and HF alarm activation had less effect on hospital admissions. In fact, HF alarms were one of the causes which least influenced hospitalizations in 2019 (OR = 9.651 CI = 2.071–44.973 *p* = 0.004). This confirms the usefulness of HMs in preventing inappropriate urgent visits.

According to previous studies, remote monitoring can reduce emergency department/urgent visits and the need of urgent care and hospitalization for HF in patients with CIEDs. [9,10]. Interestingly, in 2020 we noticed a statistically relevant increase in HF alarm activation (*p* = 0.012) compared to the control period in 2019. However, this increase did not lead to an increase in hospitalizations for HF. Probably, this increase in HF alarms was caused by the reduced daily activity of patients who were forced to stay at home during lockdown. On the one hand, sedentariness may have caused the activation of HF parameter recognition systems which are based on increased chest impedance, fluid accumulation and heart rate variability [11,12,13]. On the other hand, according to other data in the literature, we found a dramatic decrease in the number of HF hospitalizations during COVID-19 lockdown. [14,15]. We hypothesized that this is due to the need to admit only the most urgent patients into hospital. This implied that many patients hospitalized for HF at the time of admission had more severe symptoms than before the pandemic [16]. For our experience, it was pivotal to combine HM data with telemedicine. In this way we managed the majority of HF patients from home, optimizing the medical therapy for 34 patients (10.8%), avoiding inappropriate hospitalizations. Only one case, in fact, required an urgent in-hospital visit after the failure of home therapy management. Specifically, for 15 patients we modified the dosages of loop diuretics (furosemide 50 mg to 100 mg in 4 patients, 75 mg to 150 mg in 4 patients, 175 mg to 125 mg in 3 patients, 175 mg to 250 mg in 4 patients); in 11 patients we modified the dosages of ACEi/ARB/ARNI (ramipril 5 mg to 10 mg in 2 patients, perindopril 10 mg to 5 mg in 1 patient, telmisartan 40 mg to 80 mg in 2 patients, olmesartan 20 mg to 10 mg in 1 patient, 20 mg to 40 mg in 2 patients, sacubitril/valsartan 49/51 mg to 24/26 mg in 1 patient, and 49/51 mg to 97/103 mg in 2 patients); in 8 patients we changed the dosages of beta blockers (bisoprolol from 7. 5 mg to 5 mg die in 3 patients, from 2.5 mg to 3.75 mg in one patient, from 7.5 mg to 10mg in one patient, carvedilol 25 mg to 50 mg in one patient, from 12.5 mg to 25 mg in one patient, and from 75 mg to 50 mg in one patient).

Notably, not only did HF admissions decrease during the lockdown period but also hospitalizations for ACS [5,6,7,8]. Certainly, this reduction is related to the fear patients had of contracting the infection in a hospital. In addition, to avoid unnecessary hospital urgent visits, the emergency rooms have been reorganized and physicians tried to optimise home therapy before suggesting hospital access. 

It is also interesting to note that in 2020 the increase in HF alarms was associated with a reduction in almost all alarms recorded for arrhythmic events. We observed a low heart rate variability (HRV) in our patients. HRV is one of the most widely used non-invasive methods to evaluate the activity of the autonomic nervous system on the heart [17]. A low HRV seems to be related to VF, while it is not associated with stable monomorphic VT or NSVT, suggesting that the autonomic nervous system influences arrhythmia presentation [18]. A similar result was recently obtained from a multicenter study in the USA where ventricular arrhythmias requiring device therapies decreased significantly [19]. The latter may explain why we observed a decrease in non-fatal arrhythmias (such as VT, NSVT, AF), while there was no significant decrease in potentially fatal arrhythmias, such as VF, between 2019 and 2020.

Lastly, the re-modulation of the sympathetic tone is also influenced by the reduction in the levels of patient psychophysical stress. Some of the potential stress factors, such as urban traffic, work routine and metropolitan pollution, disappeared as a result of restrictive measures imposed by the Italian Government.

Our study has various limitations. First, the study was designed retrospectively. Second it has a small patient cohort from a single centre. Third, it was not possible to create a logistic regression model to evaluate the predictors of hospitalizations in 2020 due to the low number of admissions. Therefore, the results should be confirmed and validated in a larger and more representative patient cohort.

## 5. Conclusions

This study demonstrated that remote monitoring combined with telemedicine use, has ensured the surveillance of patients with cardiac devices, even when outpatient visits were not possible, for example, during the outbreak of COVID-19, avoiding unnecessary hospital assessments, managing treatment of patients from remote locations and reducing the risk of infection. During the lockdown period, arrhythmia events recorded in remote home monitoring were reduced compared to those observed in 2019, and hospital admissions for these episodes were drastically reduced. Emergency department visits for heart failure episodes also decreased in the months of the pandemic, despite the increase in heart failure exacerbations shown by HM. In 2020, patients with HF arrived in hospital in a worse clinical condition compared to months before the pandemic, while remotely managed patients avoided a worsening of clinical conditions upon admission.

## Figures and Tables

**Table 1:** Demographic characteristics and clinical features.

	*n* or Mean ± SD	%
**Population**	312	100
Female	151	48.4
Male	161	51.6
Age	71 ± 7.2	
Hypertension	194	62.1
Diabetes	87	27.9
COPD	56	18
CRF	63	20
AF/AFL	104	33.3
**Echocardiographic Data**		
EF	43.5 ± 9.8	
Diastolic Dysfunction (cumulative)	192	61.5
Grade I Grade II Grade III	118 63 11	37.8 20.2 3.5
Mitral Valve Disease	51	16.3
Aortic Valve Disease	28	8.9
Tricuspid Valve Disease	44	14.1
**Medical Therapy**		
ACEi/ARB/ARNI	262	84
Anticoagulant therapy	104	33.3
Statin therapy	123	39.4
Beta blocker	158	50.6
Antiplatelet therapy	94	30.1
Aldosterone antagonist	106	33.6
Diuretics	141	45.2
Digoxin	12	3.8
Antiarrhythmic Drugs	59	18.9
**CIEDs**		
ICD/CRT-D	127	40.7
PM	185	59.3

Data are presented as value and percent of patient group: COPD = chronic obstructive pulmonary disease; CRF = chronic renal failure (defined as eGFR < 60 mL/min/1.73 m^2^); AF/AFL = atrial fibrillation, atrial flutter; EF = ejection fraction; ACEi = angiotensin-converting enzyme inhibitors; ARB = angiotensin receptor blockers; ARNI = angiotensin receptor neprilysin inhibitors; CIEDs = cardiac implantable electronic devices; ICD = implantable cardioverter defibrillator; CRT-D = cardiac resynchronization therapy defibrillator; PM = pacemaker.

**Table 2:** Remote Monitoring Event Analysis.

	2019		2020		
**Event**	***N***	**%**	***n***	**%**	***p*-Value**
VF	4	1.3	2	0.6	0.687
NSVT	57	18.3	31	9.9	**0.002**
SVT	11	3.5	0	0	
ATP	5	1.6	8	2.6	0.581
DC Shock	1	0.3	0	0	
A-Shock	3	1	0	0	
I-Shock	0	0	0	0	
AF	91	29.2	70	22.4	**0.019**
HF Alarm	55	176	79	25.3	**0.012**
VLNA	6	1.9	14	4.5	0.115
ALNA	23	7.4	22	7.1	1.000
ERI	6	1.9	1	0.3	0.125
PMT	16	5.1	18	5.8	0.791
Hospitalization	20	6.4	2	0.6	**<0.001**

VF = ventricular fibrillation, NSVT = non-sustained ventricular tachycardia, SVT = sustained ventricular tachycardia, ATP = anti-tachycardia pacing therapy, A-Shock = appropriate shocks therapy, I-Shock = inappropriate shocks therapy, AF = atrial fibrillation, HF Alarm = heart failure alarm activation, VLNA = ventricular lead noise alert, ALNA = atrial lead noise alert, ERI = elective replacement indicator battery alarm, PMT = pacemaker-mediated tachycardia. Significant results are in bold.

**Table 3:** 2019 Binary Logistic Regression of hospitalizations.

Event	*n* (%)	S.E.	*p*-Value	OR	CI (95%)
VF	4 (1.3)	1.606	**0.001**	**262.444**	11.26–6114.34
NSVT	57 (18.3)	0.738	**0.017**	**5.788**	1.36–24.60
SVT	11 (3.5)	1.101	**0.001**	**39.264**	4.53–339.94
AF	91 (29.2)	0.791	**0.004**	**9.667**	2.05–45.55
HF Alarm	55 (17.6)	0.785	**0.004**	**9.651**	2.07–44.97
VLNA	6 (1.9)	1.161	**<0.001**	**66.909**	6.88–650.66
ALNA	23 (7.4)	1.173	**0.028**	**13.138**	1.31–130,942
ERI	6 (1.9)	1.466	**<0.001**	**827.547**	46.72–14654.91

S.E. = standard error, OR = odds ratio, C.I. = confidence interval. VF = ventricular fibrillation, NSVT = non-sustained ventricular tachycardia, SVT = sustained ventricular tachycardia, AF = atrial fibrillation, HF Alarm = heart failure alarm activation, VLNA = ventricular lead noise alert, ALNA = atrial lead noise alert, ERI = elective replacement indicator battery alarm. Significant results are in bold.

## Data Availability

Data supporting reported results can be found by contacting the Department of Cardiology of Policlinico Tor Vergata, Rome, Italy, Tel.: +39-06-2090-4044.
